# Family-based activity settings of children in a low-income African context

**DOI:** 10.4102/ajod.v8i0.364

**Published:** 2019-04-23

**Authors:** Sadna Balton, Kitty Uys, Erna Alant

**Affiliations:** 1 Center for Alternate and Augmentative Communication, University of Pretoria, Pretoria, South Africa; 2 Speech Therapy & Audiology, Chris Hani Baragwanath Academic Hospital, Soweto, South Africa; 3 Department of Occupational Therapy, University of KwaZulu-Natal, Durban, South Africa; 4 Department of Special Education, Indiana University, Bloomington, United States

**Keywords:** activity settings, culture, family, indigenous knowledge, intervention, contribute, low income, utilised, Euro-American, poor sustainability

## Abstract

**Background:**

There has been an overwhelming call to improve the understanding of how children develop within an African context as Euro-American definitions of competence have been uncritically adopted as the norm for children in Africa. The activities that children engage in within the family setting are seen as important to understand how children develop within context. The use of activity settings is closely aligned with a strengths-based perspective of family-centred practice and contributes to improved sustainability of intervention.

**Objectives:**

This study that was conducted in Soweto, South Africa, aims to describe activity settings that typically developing young children in low-income African contexts participate in.

**Method:**

A descriptive design using structured interviews was utilised to obtain information about activity settings that children aged 3–5 years and 11 months engaged in. Structured interviews with 90 caregivers were conducted.

**Results:**

Findings show that children participate in a variety of activities with varied participation levels. The types of activities are dependent on the context and perceptions of caregivers.

**Conclusion:**

These findings draw attention to understanding activities that children engage in within the family context.

## Introduction

Children within the African context have historically been judged by Euro-American definitions of competence, which have uncritically been adopted as the norm for all children (Nsamenang 2008a, 2008b; Pence, Evans & Garcia 2008; Pence & Schafer 2006). Ecocultural theory proposes that indigenous conceptions of competence should be uncovered by looking at how children are reared according to what parents know about what would be useful for their children within their specific communities (Berry 2003; Norton 1990). Various studies (Beckert, Strom & Strom 2004; Bornstein & Cote 2004; Evans, Matola & Nyeko 2008; Gaskins 1999; Geiger & Alant 2008; Rao, McHale & Pearson 2003) have shown that each culture focuses on what is valued and valid within its specific context (Serpell & Marfo 2011; Super et al. 2011). According to Ecocultural theory, development occurs along pathways determined by culture and society, and actively chosen and engaged in by parents and children, within a particular cultural ecology (Weisner 2002; Weisner et al. 2005). These pathways consist of activities and practices that are viewed as being the most important influences in the child’s and family’s life (Bernheimer & Weisner 2007). Children experience different kinds of learning opportunities, depending on where they live, what their parents enjoy doing and their values and desires for their children and families (Dunst & Bruder 1999). It is within this context that the family context provides developmental pathways for children, which are made up of the everyday routines that children engage in (Weisner 2002). The family therefore is the primary milieu in which children begin to learn the competencies expected of them within their culture and community (Britto & Ulkeur 2012; Turnbull, Turbiville & Turnbull 2000).

Activity settings which consist of the everyday experiences and events that involve the child’s interactions with various people and the environment have been recommended as the method of looking at the child within context (Farver 1999; Trivette, Dunst & Hamby 2004). Through participation in activity settings, children learn what is expected of them and learn how to determine which activities are considered appropriate or inappropriate (Tudge et al. 2013). According to Weisner (2002), parents want children to have the dispositions that would help them gain relevant skills to maintain a respectful life in their community and family. Children learn these skills through activity settings, which provide an understanding of how families structure their time, based on tradition, the socio-economic system within which they live and the orientations provided by culture (Goldenberg, Gallimore & Reese 2001; Tudge et al. 2013). Activities are therefore useful units for cultural analysis, because they are meaningful for both parents and children (Weisner 2002).

Dunst (2007) defined early childhood intervention as the experiences and opportunities afforded to children with disabilities by their parents and other caregivers that are intended to promote their competencies to shape and influence their interactions with people and objects. It therefore becomes critical for early childhood interventionists to gain insight into the activities that young children are exposed to within family settings as this influences their participation, engagement and learning.

This article describes the of activities that children living in Soweto, South Africa, participate in within the family context. The types of activities, frequency of participation and the importance of activities as rated by caregivers are discussed.

## Method

### Design

A descriptive design using structured interviews was utilised to obtain information about the activity settings that children aged 3–5 years and 11 months engaged in. Face-to-face interviews with 90 caregivers were conducted. A structured interview schedule consisting of a written list of closed-ended and open-ended questions was used. This approach was chosen as it holds no bias against participants who have varied literacy levels. Ethical clearance was obtained from the ethics committee in the Faculty of Humanities at the university.

### Setting

Participants reside in Soweto, South Africa. Soweto is a large residential urban area where a diverse group of African cultural groups reside. This city has seen rapid development and transformation over the past few years (Phadi & Ceruti 2011). However, a large proportion of Soweto’s residents remain unemployed (Patel 2012) and accommodation is mostly limited to small houses with limited space (Seekings 2000). The extended family system, which consists of multi-generational female-headed households, is still the most prevalent structural family form in Soweto (Moeno 2006).

### Sampling

Permission to conduct the study at African Self-Help Association’s (ASHA) crèches in Soweto, South Africa, was obtained in writing from the director of ASHA. Four crèches were randomly selected from a group of 40 crèches run by a non-governmental organisation in Soweto. Consent letters were sent to the caregivers of children who met the selection criteria of being between 3 and 5 years and 11 months with no known disability. Typically developing children were identified by the ASHA’s inclusion coordinator who is responsible for developmental screening at the crèches. Participants were selected through a stratified sampling procedure that accounted for age, gender and development. Ninety participants were assigned to groups of children from three-, four- and five-year age groups with equal gender representation among the children.

### Participants

The majority (56%, *n* = 50) of participants were mothers, followed by grandmothers (20%, *n* = 20), fathers (10%, *n* = 9) and others which included cousins, siblings and neighbours (10%, *n* = 9) and aunts (4%, *n* = 4). The age of participants ranged from 16 to 72 years, with 52% of participants being under 32 years, 22% between 35 and 45 years, 17% between 46 and 59 years and 9% over 60 years. Of the participants, only 20% completed higher education; 38% completed matric, which is the final year of high school in South Africa; 31% completed secondary school; 10% completed senior primary; and 1% completed junior primary. The monthly income of the majority (75%) of families was below the minimum individual taxable income of R4500.00 (USD $67.79) per month (South African Revenue Services [SARS] 2008). An average of 5.6 family members share a four-room house.

### Description of interview schedule

The interview schedule was based on the Parent Survey of Home and Family Experiences (Dunst & Bruder 1999). Permission to use the survey was obtained in a face-to-face meeting with one of the authors. The survey was adapted for the South African context through an expert panel and focus group discussions (Balton 2009). The expert panel consisted of three speech therapy assistants employed at a hospital in Soweto. The speech therapy assistants have over 20 years of experience each in working with families from the Soweto community and also live within the same community as the target population. The interview schedule (Appendix 1) included a list of closed-ended and open-ended questions. Part 1 included biographical information and part 2 contained 50 closed-ended questions relating to activities that children participated in. The following eight main categories of activities were included: (1) child routine activities (family mealtimes, bathing, dressing and undressing, toileting, washing hands, brushing teeth, haircut or styles, visiting the local clinic and carried on the back); (2) play activities (running, jumping and chasing, playing with toys, pretend games, lap games, playing with water, playing with sand, hand or finger games, ‘mokuku’ hide-and-seek, building blocks, arcade games and cell phone games); (3) early literacy activities (having a conversation, telling stories, listening to stories, reading or looking at books, colouring, drawing, painting, cutting and pasting); (4) entertainment activities (watching television, singing, listening to music and dancing); (5) chores (cleaning the yard, washing socks and underwear, setting the table, assisting with preparing meals and gardening); (6) spiritual activities (attending church, praying, attending an ancestral ceremony and attending funerals); (7) family activities (family gatherings, visiting family or friends in the neighbourhood and visiting the family or traditional home); (8) community activities (visiting shopping malls, eating out, going to the ‘spaza’ informal shop, attending parties, attending weddings, taxi rides and visiting a park).

In part 2 of the interview, participants were asked to comment on the frequency of participation and reasons for non-participation and to identify the partners involved in the activity with the child and state the main purpose of the activity. The participants were also required to rate each activity in terms of its importance for learning on a scale of 1–3, with 1 = not important, 2 = important and 3 = very important. Part 3 of the interview was composed of four open-ended questions to obtain insight into caregiver beliefs and perceptions about activities.

### General procedures

#### Data collection procedures

The scripted, structured face-to-face interviews were conducted by the researcher at one of three crèches. The interviews were audio-taped and took approximately 35 minutes to complete. The researcher commenced the interview by stating its purpose and allowing participants time for questions. Instructions were read out exactly as they appeared on the interview schedule, following a sequential order of questions and using the same material for all interviews (Mathers, Fox & Hunn 2002). The responses were recorded directly on the interview schedule. The interview concluded with the interviewer thanking the participant and allowing time for any further questions about the study.

### Reliability of data

Thirty per cent of the interviews were randomly selected by two speech-language therapists who checked the reliability of the recording and the coding of information (McMillan & Schumacher 2001). Inter-rater agreement of 100% was achieved.

### Data analysis

The data were analysed using both qualitative and quantitative methods. Descriptive statistical procedures, in particular frequency tables, were used to organise the data collected. The results were quantified in terms of means, standard deviation, frequencies and relationships between variables. A content analysis procedure was conducted on responses to open-ended questions in order to identify common categories that were then utilised to establish codes.

### Ethical considerations

Ethical clearance was obtained from the ethical committee at the University of Pretoria, reference number: 21277177.

## Results

Table 1 shows the percentage participation rate in activities as well as the frequency (daily, weekly, monthly and annually or none) of participation. Participation levels were subjectively divided into high (80% – 100%), moderate (50% – 79%) and low (< 50%). High daily participation was recorded for family meals (100%), bathing (100%), dressing and undressing (100%), toileting (100%), washing hands (100%), brushing teeth (99%), having a conversation (95%), watching television (92%) and listening to music (87%). Moderate daily participation rates were indicated for playing with toys and praying (78%), singing (72%), dancing (57%), colouring and pretend games (52%), playing with water and reading and looking at books. High weekly participation rates were recorded for attending church (72%) and visiting shopping malls (56%). Moderate monthly participation rates were indicated for attending parties (67%), having a haircut (61%), eating out (59%), family gatherings and going to the clinic (52%).

**TABLE 1:** Frequency of participation (*n* = 90).

Category	Activity	Frequency (*n*)	Participation in activity (%)	Daily	Weekly	Monthly	Annually	Never
**Child routine activities**	Family meals	90	100	100	-	-	-	-
	Bathing	90	100	100	-	-	-	-
	Dressing and undressing	90	100	100	-	-	-	-
	Toileting	90	100	100	-	-	-	-
	Washing hands	90	100	100	-	-	-	-
	Brushing teeth	89	99	99	-	-	-	1
	Haircut	89	99	7	30	61	1	1
	Carried on the back	67	74	31	21	21	1	26
**Play**	Running, jumping	89	99	90	9	-	-	1
	Playing with toys	89	99	78	15	7	-	-
	Pretend games	82	91	52	26	13	-	9
	Playing with water	75	83	50	19	14	-	17
	Lap games	75	83	51	19	13	-	17
	Hand or finger games	71	79	26	34	18	1	21
	Playing arcade games	70	78	-	22	3	55	20
	Playing with blocks	62	68	14	40	14	-	32
	Riding a bike or scooter	59	66	32	26	8	-	34
	Play with sand	59	66	28	24	13	1	34
	‘Mokuku’ hide-and-seek	58	64	21	22	21	-	36
	Cell phone games	44	49	20	17	11	1	51
**Early literacy**	Having a conversation	89	99	95	2	2	-	1
	Reading or looking at books	83	92	50	32	10	-	8
	Colouring	82	91	52	28	11	-	9
	Telling stories	79	88	33	29	26	-	12
	Listening to stories	77	86	36	33	17	-	14
	Cutting and pasting	69	77	26	30	21	-	23
**Entertainment**	Watching TV	90	100	92	7	1	-	-
	Singing	85	94	70	13	11	-	6
	Dancing	83	92	57	-	-	29	14
	Listening to music	82	91	87	13	-	-	-
**Spiritual**	Praying	84	93	78	8	7	-	7
	Church	82	91	5	72	13	1	9
**Chores**	Cleaning the yard	48	53	4	31	18	-	47
	Washing socks and underwear	44	49	26	14	9	-	51
	Setting the table	38	42	18	7	15	2	58
	Assisting with preparation of meals	37	41	11	12	17	1	59
	Gardening	35	39	2	22	15	-	61
**Family**	Family gatherings	88	98	2	16	53	27	2
	Eating out	87	97	-	38	59	-	3
	Visiting family and friends	78	87	21	37	27	2	13
	Visiting family or traditional home	67	74	21	37	27	2	13
**Community**	Visiting shopping malls	90	100	-	56	43	1	-
	Visiting a community clinic	82	91	-	1	52	38	9
	Attending parties	79	88	-	2	67	19	12
	Taxi ride	77	86	2	44	36	4	14
	Going to the ‘spaza’ (informal) shop	67	74	38	22	14	-	26
	Visiting a park	59	66	5	13	30	18	34
	Attending weddings	49	54	-	-	24	30	46
	Attending an ancestral ceremony	35	39	3	3	17	16	61
	Attending funerals	19	21	0	0	7	14	79

Low participation rates (< 50%) were reported for attending weddings (49%), cleaning the yard (48%), washing socks and underwear and cell phone games (44%), setting the table (38%), assisting with preparation of meals (37%), attending an ancestral ceremony and gardening (35%) and attending funerals (19%).

## Caregiver perceptions of activities that are important for learning

Caregivers were asked to rate the importance of activities for learning by stating if an activity was very important, important or not important. The results displayed in Table 2 indicate the mean score obtained, with the maximum being 3 and the minimum 1. Activities rated as very important have a mean of 2.5–3.0, activities rated as important have a mean of 2.00–2.49 and not important has a mean score *of* < 2 (see Table 2).

**TABLE 2:** Caregiver ratings on the importance of activities for learning.

Category	Activity	Very important		Important		Not important	
		Mean	SD	Mean	SD	Mean	SD
**Child routines**	Washing hands	2.68	0.46	-	-	-	-
	Mealtimes	2.66	0.47	-	-	-	-
	Visiting the clinic	2.64	0.48	-	-	-	-
	Toileting	2.61	0.49	-	-	-	-
	Dressing and undressing	2.56	0.49	-	-	-	-
	Brushing teeth	2.56	0.49	-	-	-	-
	Bathing	2.55	0.49	-	-	-	-
	Haircut or style	-	-	2.31	0.53	-	-
	Carried on the back	-	-	2.02	0.69	-	-
**Play**	Playing with toys	-	-	2.44	0.62	-	-
	Playing with blocks	-	-	2.39	0.49	-	-
	Running, jumping, chasing	-	-	2.33	0.58	-	-
	Pretend games	-	-	2.26	0.58	-	-
	Arcade games	-	-	2.22	0.58	-	-
	Cell phone games	-	-	2.22	0.64	-	-
	Lap games	-	-	2.20	0.59	-	-
	Hand or finger games	-	-	2.19	0.49	-	-
	Riding a bike or scooter	-	-	2.16	0.56	-	-
	‘Mokuku’ hide-and-seek	-	-	2.05	0.57	-	-
	Playing with water	-	-	-	-	1.88	0.67
	Playing with sand	-	-	-	-	1.66	0.72
**Early literacy**	Colouring, drawing, painting	2.74	0.43	-	-	-	-
	Having a conversation	2.68	0.46	-	-	-	-
	Reading or looking at books	2.65	0.47	-	-	-	-
	Listening to stories	2.62	0.51	-	-	-	-
	Cutting and pasting	2.62	0.51	-	-	-	-
	Telling stories	2.53	0.50	-	-	-	-
**Entertainment**	Watching television	-	-	2.47	0.54	-	-
	Singing	-	-	2.36	0.59	-	-
	Dancing	-	-	2.14	0.58	-	-
	Listening to music	-	-	2.14	0.61	-	-
**Chores**	Washing socks or underwear	-	-	2.47	0.50	-	-
	Assisting with setting the table	-	-	2.47	0.55	-	-
	Preparing meals	-	-	2.43	0.60	-	-
	Cleaning the yard	-	-	2.35	0.63	-	-
	Gardening	-	-	2.31	0.47	-	-
**Spiritual**	Attending church	2.74	0.45	-	-	-	-
	Praying	2.66	0.47	-	-	-	-
	Attending ancestral ceremony	-	-	2.40	0.49	-	-
	Attending funerals	-	-	2.05	0.40	-	-
**Family**	Family gatherings	-	-	2.46	0.54	-	-
	Visiting the traditional home	-	-	2.34	0.56	-	-
	Visiting family friends	-	-	2.15	0.58	-	-
**Community**	Visiting a park	-	-	2.47	0.56	-	-
	Eating out	-	-	2.22	0.56	-	-
	Shopping malls	-	-	2.22	0.59	-	-
	Spaza shop	-	-	2.14	0.58	-	-
	Taxi ride	-	-	2.12	0.67	-	-
	Attending weddings	-	-	2.12	0.59	-	-

SD, standard deviation.

The categories rated as very important for learning include most child routine activities and all early literacy activities, with colouring, drawing and painting (mean = 2.74, SD = 0.43) rated the most important of all activities. Other activities in this category include having a conversation (mean = 2.68, SD = 0.46), reading or looking at books (mean = 2.65, SD = 0.47), listening to stories (mean = 2.62, SD = 0.51), cutting and pasting (mean = 2.61, SD = 0.51) and telling stories (mean = 2.53, SD = 0.50). Spiritual activities rated as very important consisted of attending church (mean = 2.74, SD = 0.45) and praying (mean = 2.66, SD = 0.47). All activities in the entertainment, chores and family category were rated as important for learning. Most play activities except for playing with water and playing with sand were rated as important for learning. Two activities from the play category, namely playing with water and playing with sand, were rated as not important for learning.

Caregivers were also requested to state what they viewed as the main purpose of an activity according to the following categories: fun, work, socialisation, care, educational, exercise, spiritual and other. The percentage was calculated for each category (see Table 3).

**TABLE 3:** Caregivers perceptions about the purpose of activities (*N* = 90).

Activity	Fun	Work	Socialisation	Care	Educational	Exercise	Spiritual	Other
Family meals	18	1	25.0	31	21	2	-	2
Bathing	15	3	3.0	42	29	8	-	-
Brushing teeth	10	4	-	33	46	7	-	-
Dressing and undressing	1	2	1.0	29	56	11	-	-
Toileting	2	1	-	35	49	12	-	1
Washing hands	9	-	-	33	49	8	1.0	-
Haircut	11	1	7.0	73	6	-	2.0	-
Attending the clinic	1	-	-	83	11	1	1.0	3
Carried on back	33	-	4.0	45	3	12	3.0	-
Playing toys	34	1	18.0	3	26	17	1.0	-
Play with sand	58	6	6.0	2	14	14	-	-
Play with water	75	1	1.0	4	6	12	-	1
Cell phone games	39	-	2.0	2	46	11	-	-
Arcade games	56	-	16.0	1	13	14	-	-
Pretend games	36	1	2.0	5	43	12	1.0	-
Riding a bike or scooter	48	-	5.0	-	8	39	-	-
Mokuku	55	-	12.0	2	14	17	-	-
Blocks	14	5	6.0	2	55	18	-	-
Hand or finger games	40	-	4.0	6	20	30	-	-
Lap games	23	-	11.0	39	4	20	2.0	1
Running, jumping and chasing	30	1	2.0	6	10	51	-	-
Having a conversation	6	2	36.0	7	46	-	3.0	-
Listening to stories	10	-	9.0	7	65	5	4.0	-
Telling stories	15	3	14.0	5	58	4	1.0	-
Reading or looking at books	10	2	2.0	1	77	8	-	-
Colouring, drawing and painting	6	3	5.0	1	74	11	-	-
Cutting and pasting	6	9	-	3	71	11	-	-
Cleaning yard	27	17	6.0	6	25	17	2.0	-
Washing socks or underwear	20	14	-	18	32	16	-	-
Preparing meals	19	5	8.0	3	51	11	-	3
Setting the table	8	8	2.5	16	42	21	2.5	-
Gardening	14	17	-	6	40	23	-	-
Visiting shopping malls	39	-	20.0	12	21	6	1.0	1
Going to the spaza shop	17	9	9.0	9	28	28	-	-
Eating out	38	-	29.0	21	8	1	2.0	1
Attending weddings	29	-	45.0	12	10	2	2.0	-
Attending parties	51	-	42.0	4	2	-	1.0	-
Taking a taxi	26	1	12.0	13	26	16	1.0	5
Park	41	-	20.0	10	19	8	2.0	-
Listening to music	39	-	11.0	1	33	9	7.0	-
Dancing	43	-	12.0	4	6	35	-	-
Singing	35	-	4.0	-	33	12	16.0	-
Watching TV	18	-	10.0	-	70	2	-	-
Attending church	1	4	4.0	-	18	-	73.0	-
Attending an ancestral ceremony	-	-	14.0	9	31	-	46.0	-
Attending funerals	-	-	5.0	11	16	-	63.0	5
Praying	4	-	-	4	33	1	58.0	-
Family gatherings	13	-	59.0	10	9	-	-	9
Attending weddings	29	-	45.0	12	10	2	2.0	-
Visiting family or friends	19	-	51.0	13	10	4	3.0	-
Visit traditional home	13	2	45.0	15	13	2	10.0	-

Results show that most activities from the play and entertainment categories were perceived as fun, while family activities were considered to serve the purpose of socialisation. Only four activities were perceived care, as these were from the child routine and community categories. Activities identified as educational were mainly from the early literacy and child routine categories. No play activities were highly rated as educational. All activities in the spiritual activity category were considered as having a spiritual purpose. An insignificant percentage of activities were seen as work or being done for the purpose of exercise.

## Open-ended questions

The first open-ended question aimed at determining if there were any activities that the children participated in which were not included in the questionnaire. Of the participants, 54% did not add any further activities and 24% added play activities which included soccer, wrestling and basketball. The second open-ended question explored what participants considered as important lessons that children should learn from home. Fifty per cent of participants identified morals and values as important lessons, 48% identified self-care and hygiene while only 22% stated that educational and literacy activities were important lessons from home. The third open-ended question surveyed participants’ perceptions on what activities the child enjoyed most at home. Of the participants, 80% stated that children enjoyed playing, 58% listed entertainment and social activities and 56% stated that children enjoyed singing and dancing. The forth open-ended question required the respondents to share their perceptions on how children learnt, from which four themes were identified. Fifty-three per cent of participants stated that children learnt best by participating in activities, 27% stated through spending time with family, 19% stated by being at a crèche and 12% stated through observation.

## Discussion

Families provide a rich cultural context in which children learn and develop (Carpenter 2000). Gaining insight into children’s activity settings within the family context is important as families prefer intervention approaches that can easily be incorporated into their daily lives (Sheldon & Rush 2001) and are congruent with their beliefs and practices. The discussion will focus on the frequency of participation in activities within the eight categories identified earlier as well as how these activities provide an opportunity for learning within the family context.

Child routine activities, which are mainly essential for daily care, have the highest weekly participation rates and were rated by participants as very important for learning (see Table 2). This is consistent with the literature, as Dunst, Meter and Hamby (2001) suggest that the repetitiveness and frequency of occurrence of routine activities provide children with an opportunity to learn and practise new skills within context. The importance of self-care and hygiene was stated as an important lesson from home and seen by most participants as having an educational purpose. These activities also allow children to gain insight into family culture. Larson and colleagues explain that mealtime is seen as a vehicle of culture because ‘through mealtime activities and conversation, family members often enact and reaffirm cultural meanings and create new meaning’ (Larson, Branscomb & Wiley 2006:3). Jarret, Bahar and Kersh (2014) showed that low-income African American caregivers showed that they valued family mealtimes and acknowledged the benefits for family life. Various studies have shown the benefits of family mealtimes; this activity provides an opportunity for children to learn new words in context (Beals 1997), for parents to listen to children talk about their daily lives (Fulkerson et al. 2010) and has also been associated with enhancing family cohesion and contributing to positive developmental outcomes. A study conducted by De Grace et al. (2016) showed the benefits of improved social and family outcomes for children with special therapeutic and behavioural needs. The positive benefits of family mealtimes highlight the need for early childhood interventionists to look at strategies to increase the participation of children with disabilities in this key family activity.

The role of play in child development has historically been applied by looking at it through the lens of western cultures. According to Roopnarine and Davidson (2015:231), play is ‘culturally situated, and mothers and fathers support play in multiple ways across cultures and time’ This depends on how the community is structured, how play is defined and the kind of significance attributed to play by the community (Göncü et al. 1999). Children in this study most frequently engage in running, jumping and chasing, playing with toys and pretend games. Participants viewed most play activities as having a fun purpose for children. These results correlate with a study of 127 families across 28 developing countries which found that taking children outdoors and play were the most predominant activities that children were involved in (Bornstein & Putnick 2012). Pretend play themes are often linked to culture (Nielsen, Cucchiaro & Mohamedally 2012) as different cultural groups may engage in pretend play for different purposes and play themes may vary according to children’s settings (Göncü et al. 1999). Participants in the focus groups that were part of the preparatory phase of this study stated that children liked pretending to be a mother by tying a doll on their backs, or being a taxi driver or a teacher. These are the roles that children are regularly exposed to in their daily settings. Furth (1996) showed how cultural practices link to pretend play in a township in Durban, South Africa, where children were pretending to slaughter a cow which is a ‘real-world’ activity that is transferred into children’s pretend world (Göncü et al. 1999). Results indicate that children frequently participate in water play which is seen as having a fun purpose, and was rated as not important for learning. Caregiver views on water play may be related to the fact that water is considered an expensive commodity in South Africa. Water is free up to 6000 L per household and usage is monitored by pre-pay water metres (Ruiters 2007). This resource is also shared by large families and sometimes by more than one family and is therefore unlikely to be used in play activities. The lower frequency of play with sand could be that most families do not keep gardens because it is expensive to maintain because of the cost of water and that access to sand may be limited because of lack of space (Balton 2009). Early interventionists need to explore alternate activities within the family context that can provide children with alternate sensory experiences that sand and water play would expose them to. This could include activities such as assisting with washing vegetables for cooking, helping to measure and mix ingredients during baking and making a fruit salad to explore different textures.

Colouring, drawing and pasting was rated as the most important activity for learning; this is an interesting ranking as it was seen as more important than reading or looking at books and telling stories. This perception may be based on the nature of activities that children take home from school or that colouring and drawing may not require adult supervision. Children engage in conversation on a daily basis, and the topics of these conversations include what they did at school, details of their play with friends and discussions of what they watched on television (Balton 2009). Telling stories occurs less frequently than listening to stories, which has been identified as a means for family history to be shared with young children, thus providing an avenue for values to be imparted (Sameroff & Fiese 2000). Participants in the focus group phase of this study stated that grandmothers often told children stories about their past to teach children lessons and for them to learn about their family’s history (Balton 2009). According to Ouduaran (2013), in African culture, grandmothers often teach younger generations about African wisdom and culture through storytelling.

Children’s participation in entertainment activities is important for literacy development as it increases children’s ability to shape and understand the available meanings in any number of expressive systems including the media, the arts and popular culture (Dills 2007; Eisner 1998). Children’s high participation rate for watching television suggests that this activity plays a significant role in their daily experiences. Results also showed that 70% of the participants perceive watching television as having an educational purpose. Children are allowed to watch television because it is believed to improve their English as well as their concentration, and that it is much safer than playing outdoors (Balton 2009). This sentiment on safety was echoed by Jordan (2005) who interviewed 42 families who live in high-crime areas, where watching television was seen as a safe and relatively inexpensive way of keeping young children occupied. Burdette and Whitaker (2005) also found in a sample of 20 large cities in in the United States (US) that mothers’ perceptions of neighbourhood safety impacted on children’s viewing time. Their findings showed that children who lived in neighbourhoods that were perceived as unsafe watched more television.

Children’s high participation in singing, dancing and listening to music could be ascribed to the fact that music and music making is an inherent part of South African culture which assists in the transmission of its cultural heritage (Woodward 2007). These activities, especially music, are highly accessible in daily life in varied settings (Getz et al. 2011). Participants viewed singing and listening to music as fun and educational; one of the participants in the focus group stated that he got his child to sing the national anthem to learn about his country (Balton 2009). This is important as researchers in the field of early literacy have realised that promoting literacy at home no longer means recreating academic settings within the home but rather taking advantage of opportunities that arise in daily life to help children’s transition towards literacy (Cutspec 2006). Interventionists need to take cognisance of these activities as potential avenues for facilitating early literacy, because a high number of children frequently engage in them and because participants identified the educational worth of these activities.

Religion and spirituality play an important role in children’s lives and are vital to family relationships (Bartowski, Xu & Levin 2008) and in African traditional practice, religion is integral to people’s cultural background (Van Rensburg et al. 2013). Very high daily participation rates for praying and attending church weekly are shown in Table 2. Participants in the focus groups reported that children attend church to learn about their religion to become good Christians, to learn how to pray and to be thankful to God for what they have (Balton 2009). Religious activity is also reported to increase children’s resilience and provide a sense of coherence within the family (Bartowski et al. 2008; Mercer 2006; Werner 2000). Most participants identified morals and values as important lessons from home, which ties in with the high participation rate for spiritual activities.

Community life also provides children with a range of experiences in the contexts of family outings, community celebrations and other community activities (Dunst 2001). In recent years, visiting shopping malls has rapidly become an important and valuable ‘cultural’ form which is popularly seen as a mixture of convenience and leisure (Murray 1997). The accessibility of shopping malls to residents of Soweto has increased over the past 5 years, with two major malls built in 2005 and another three in 2007 (Mazibuko 2007). Visits to shopping malls are linked to participation in other activities like playing arcade games and eating out. Participants in the focus groups stated that they prefer to take children to the shopping malls because they were safer than other spaces like community parks. The high participation rate for visiting shopping malls highlights the lack of safe spaces for children to play in communities.

The activity that most children participate in at least once a month is going to the ‘spaza shop’, which is a home-based enterprise often within walking distance of children’s homes (Ligthelm 2005). This errand is reported to provide children with opportunities to learn about the environment, the dynamics of interacting with others and offers the opportunity to practise being helpful and responsible, which are important lessons in African culture (Nsamenang 1992).

The lower participation levels for being carried on the back were attributed to the age of children in this study. The reasons provided by participants for children’s lower participation levels for riding a bike or scooter and playing with blocks are ascribed to the lack of money to purchase these toys (Balton 2009). Children’s participation in playing arcade games, which are relatively expensive, highlights caregiver’s concerns related to safety as these games are played in a contained area under adult supervision. The findings of studies conducted in Australia by Carver, Timperio and Crawford (2008) and Veitch et al. (2006) and in South Africa by Kruger and Chawla (2005) concur with this statement. Their research concluded that parents’ issues about the safety of their children playing in places other than their own yard were influenced by concerns surrounding strangers, gangs and road traffic. The physical settings have also influenced children’s participation in ‘mokuku’ (hide-and-seek) as caregivers stated concerns about safety and lack of space.

Many shopping malls have been built in Soweto over the past 5 years; this has increased the availability of fast food outlets with most children eating out at least once a month. The mean score for taxi rides also indicates that most children travel by taxi once a month, which relates to the results which show that activities away from home and which require more money occur less frequently. Other activities which children participate in at least once a month include attending parties and family gatherings. While children’s participation in chores in parts of Africa have historically been an expected activity (Nsamenang 1992), results showed that the only chore activity that most children participate in is washing socks and underwear. Children’s participation in chores and work-related activities can be attributed to a number of factors and identifying a causal relationship is not possible for this complex matter. Poverty, social and economic factors, children’s rights, family size, female-headed households and whether it is an urban or rural community are some of the factors identified, which have impact on children’s participation in work-related activities (Cummings 2016). The reason for children’s low participation in chores could be attributed to the young age group investigated in this study, the larger family structure where other family members take on the responsibilities or that children spend a large proportion of their time at crèche.

Children hardly ever participated in chores like cleaning the yard and family activities such as visiting the family or traditional home. While participation levels were high for religious activity, it was much lower for traditional practices such as ancestral ceremonies and attendance at funerals. This could be because key traditional practices have been replaced by modern ones (Evans, Matola & Nyeko 2008). Less frequent visits are also conducted to the family or traditional home with participants stating that children visit at least once a year. This is understandable within the context of urbanisation, often implying that the traditional home is far from where families live and visiting would therefore incur expenses that the family may not be able to afford. Children’s visits to the park were reported as less frequent, which could be because of the fear of exposure to drugs, violence, vandalism and parents having less time available because of various stressors (Milteer & Ginberg 2012). Participants in the focus group stage of the study stated that they did not consider parks as safe for children as they were not clean and often had broken glass on the field and that there were possible criminal and drug-related activities taking place at these places (Balton 2009).

## Conclusion

Participants in this study believe that children learn most by participating in activities and by observing others. The results show that children are exposed to different types of activities and experiences depending on the beliefs, values, practices and resources of families. This was highlighted by children’s high participation in care, play and spiritual activities as well as lower participation in certain chores and educational activities like cutting and pasting. Participation in activities is also determined by access to resources (water and sand play and eating out), the lack of safety and security, which has possibly affected activities like increased visits to shopping malls and decreased visits to parks. Interventionists need to develop an understanding of family activities and integrate developmental goals within these.

This study has assisted in building on the ‘indigenous’ knowledge base of children and families in an African context, thus heeding the call to increase the knowledge base ‘about Africa for Africa’ (Pence et al. 2008). The use of activity settings is closely aligned to the strengths-based perspective of family-centred practice. The findings have increased the knowledge base regarding children within their natural environments, with these environments being rich in opportunities for learning; furthermore, the findings also contribute to (Pence & Marfo 2008):

… development of a science of child development that is not narrowly constructed on the lives of a small minority of the world’s children, but rather a science that opens up to other populations and other possibilities. (p. 85)

This study was conducted in a South African urban setting and cannot necessarily be generalised to other African contexts.

## Appendix 1: Interview schedule.

**Table:**

**Questionnaire no:**	V1	
**School:**	V2	
**Date:___________**		

**I am going to ask you a few questions about your child, yourself and your family. Please let me know if you need me to repeat or explain any of the questions**.

### Part 1: Biographical information

1. **How old is your child?**
  **Table:**
  1	3.0–3.11 years
  2	4.0–4.11 years
  3	5.0–5.11 years
  **Table:**
  V3	
2. **Is your child a boy or girl?**
  **Table:**
  1	Male
  2	Female
  **Table:**
  V4	

### Part 2: Caregiver information

3. **What is your relationship with the child? (How are you related to the child?)**
  **Table:**
  1	Mother
  2	Father
  3	Grandmother
  4	Aunt
  5	Other
  **Table:**
  V5	
4. **How old are you? ___________________ years**
  **Table:**
  V6	
5. **What standard or grade did you complete at school? Did you study further?**
  **Table:**
  1	No formal schooling
  2	Junior primary grades 1–3
  3	Senior primary grades 4–7
  4	High school grades 8–11
  5	Matric
  6	Higher education
  7	Other – specify
  **Table:**
  V7	
6. **Are you working? (If yes) Are you working full time, part time or as a casual?**
  **Table:**
  1	Employed full time
  2	Employed part time
  3	Employed casual
  4	Unemployed
  5	Other – specify
  **Table:**
  V8	
7. **What is your family’s monthly income? ________________**
  **Table:**
  V9	
8. **Who else is living in your house?**
  **Table:**
  1	Mother
  2	Father
  3	Grandmother
  4	Grandfather
  5	Great grandmother
  6	Great grandfather
  7	Brothers and sisters
  8	Aunt
  9	Uncle
  10	Cousin
  11	Other specify
  **Table:**
  V10	
  V11	
  V12	
  V13	
  V14	
  V15	
  V16	
  V17	
  V18	
  V19	
  V20	
9. **What is the total number of people in your house? _______________**
  **Table:**
  V21	
10. **How many rooms are there in your house? ______________rooms**
  **Table:**
  V22	
11. **Activity settings: Please listen carefully to the following questions:**
  If you need me to explain or repeat anything, please ask. I am going to ask you questions about activities that your child may be involved in. There are five questions related to each activity. I will ask the questions one at a time. I will show you the possible responses and a sheet to help you remember the different options for answering.
  **Table:**
  **No.**	**Activity**	**11.1 Does your child participate in this activity?** 1 – Never 2 – Hardly ever (once a year) 3 – Sometimes (once a month) 4 – Often (once a week) 5 – Daily (everyday) 11.2 If not, is it because of? 1 – Money 2 – Transport 3 – Space 4 – Time 5 – Safety 6 – Child’s age 7 – Other				**11.3. With whom does your child mainly participate with in this activity?** 1 – Mother 2 – Father 3 – Parents 4 – Siblings 5 – Family 6 – Grandparents 7 – Friends 8 – None 9 – Other		**11.4 What is the main purpose (reason) of this activity?** 1 – Fun 2 – Work or chores 3 – Socialisation 4 – Care 5 – Educational 6 – Exercise 7 – Spiritual 8 – Other		**11.5 How important do you think this activity is for your child’s learning? Please rate from 1–3** 1 = Not important 2 = Important 3 = Very important	
  1	Family meals	V23		V73		V123		V173		V223	
  2	Bathing	V24		V74		V124		V174		V224	
  3	Brushing teeth	V25		V75		V125		V175		V225	
  4	Dressing and undressing	V26		V76		V126		V176		V226	
  5	Toileting	V27		V77		V127		V177		V227	
  6	Assist in preparing meals	V28		V78		V128		V178		V228	
  7	Setting the table	V29		V79		V129		V179		V229	
  8	Washing hands	V30		V80		V130		V180		V230	
  9	Cleaning the yard	V31		V81		V131		V181		V231	
  10	Washing socks and underwear	V32		V82		V132		V182		V232	
  11	Haircut or style	V33		V83		V133		V183		V233	
  12	Watching TV	V34		V84		V134		V184		V234	
  13	Listening to music	V35		V85		V135		V185		V235	
  14	Dancing	V36		V86		V136		V186		V236	
  15	Singing	V37		V87		V137		V187		V237	
  16	Praying	V38		V88		V138		V188		V238	
  17	Having a conversation	V39		V89		V139		V189		V239	
  18	Listening to stories	V40		V90		V140		V190		V240	
  19	Telling stories	V41		V91		V141		V191		V241	
  20	Reading or looking at books	V42		V92		V142		V192		V242	
  21	Colouring, drawing, painting	V43		V93		V143		V193		V243	
  22	Playing with toys	V44		V94		V144		V194		V244	
  23	Cell phone games	V45		V95		V145		V195		V245	
  24	Cutting and pasting	V46		V96		V146		V196		V246	
  25	Playing with sand	V47		V97		V147		V197		V247	
  26	Playing with water	V48		V98		V148		V198		V248	
  27	Visiting shopping malls	V49		V99		V149		V199		V249	
  28	Playing arcade games	V50		V100		V150		V200		V250	
  29	Going to the ‘spaza’ shop	V51		V101		V151		V201		V251	
  30	Pretend games	V52		V102		V152		V202		V252	
  31	Riding a bike or scooter	V53		V103		V153		V203		V253	
  32	Mokuku	V54		V104		V154		V204		V254	
  33	Building blocks	V55		V105		V155		V205		V255	
  34	Hand or finger games	V56		V106		V156		V206		V256	
  35	Lap games	V57		V107		V157		V207		V257	
  36	Carried on back	V58		V108		V158		V208		V258	
  37	Running, jumping and chasing	V59		V109		V159		V209		V259	
  38	Eating out	V60		V110		V160		V210		V260	
  39	Gardening	V61		V111		V161		V211		V261	
  40	Family gatherings	V62		V112		V162		V212		V262	
  41	Attending weddings	V63		V113		V163		V213		V263	
  42	Attending parties	V64		V114		V164		V214		V264	
  43	Attending funerals	V65		V115		V165		V215		V265	
  44	Visiting family or friends in the neighbourhood	V66		V116		V166		V216		V266	
  45	Visit family or traditional home	V67		V117		V167		V217		V267	
  46	Attending church	V68		V118		V168		V218		V268	
  47	Attending ancestral ceremony	V69		V119		V169		V219		V269	
  48	Visiting a community clinic	V70		V120		V170		V220		V270	
  49	Taxi ride	V71		V121		V171		V221		V271	
  50	Visiting a park	V72		V122		V172		V222		V272	

### Part 3

We have come to the last part of the interview, I am going to ask you four more questions, please try to answer them all. If you need me to explain anything, please ask.

12. **Are there any other activities that your child does at home that you think he or she could learn from?**
     ____________________________________________________________
     ____________________________________________________________
     ____________________________________________________________
     ____________________________________________________________
  **Table:**
  V273	
  V274	
  V275	
  V276	
  V278	
13. **What do you (think) consider as the most important things for your child to learn at home?**
     ____________________________________________________________
     ____________________________________________________________
     ____________________________________________________________
  **Table:**
  V279	
  V280	
  V281	
  V282	
  V283	
14. **Please list, in order of importance, 3–5 home activities that make your child laugh or smile (Interesting and enjoyable).**
     ____________________________________________________________
     ____________________________________________________________
     ____________________________________________________________
  **Table:**
  V284	
  V285	
  V286	
  V287	
  V288	
15. **Please complete the following sentence; I think that my child learns best by**
     ____________________________________________________________
     ____________________________________________________________
     ____________________________________________________________
  **Table:**
  V289	
  V290	
  V291	
  V292	
  V293	

**Thank you for your participation, do you have any questions or comments**?
